# Causal influence of gut microbiota on small cell lung cancer: a Mendelian randomization study

**DOI:** 10.1111/crj.13764

**Published:** 2024-04-29

**Authors:** Wenjing Yang, Xinxia Fan, Wangshu Li, Yan Chen

**Affiliations:** ^1^ General Hospital of Ningxia Medical University Yinchuan Ningxia Hui Autonomous Region China; ^2^ The Second Affiliated Hospital of Liaoning University of Traditional Chinese Medicine Shenyang Liaoning China; ^3^ Dalian Women and Children's Medical Center (Group) Dalian Liaoning China; ^4^ Department of Respiratory and Critical Care Medicine General Hospital of Northern Theater Command Shenyang Liaoning China

**Keywords:** causality, gut microbiota, lung cancer, Mendelian randomization, small cell lung cancer

## Abstract

**Background:**

Previous studies have hinted at a significant link between lung cancer and the gut microbiome, yet their causal relationship remains to be elucidated.

**Methods:**

GWAS data for small cell lung cancer (SCLC) was extracted from the FinnGen consortium, comprising 179 cases and 218 613 controls. Genetic variation data for 211 gut microbiota were obtained as instrumental variables from MiBioGen. Mendelian randomization (MR) was employed to determine the causal relationship between the two, with inverse variance weighting (IVW) being the primary method for causal analysis. The MR results were validated through several sensitivity analyses.

**Results:**

The study identified a protective effect against SCLC for the genus *Eubacterium ruminantium* group (OR = 0.413, 95% CI: 0.223–0.767, *p* = 0.00513), genus *Barnesiella* (OR = 0.208, 95% CI: 0.0640–0.678, *p* = 0.00919), family Lachnospiraceae (OR = 0.319, 95% CI: 0.107–0.948, *p* = 0.03979), and genus *Butyricimonas* (OR = 0.376, 95% CI: 0.144–0.984, *p* = 0.04634). Conversely, genus *Intestinibacter* (OR = 3.214, 95% CI: 1.303–7.926, *p* = 0.01125), genus *Eubacterium oxidoreducens* group (OR = 3.391, 95% CI: 1.215–9.467, *p* = 0.01973), genus *Bilophila* (OR = 3.547, 95% CI: 1.106–11.371, *p* = 0.03315), and order Bacillales (OR = 1.860, 95% CI: 1.034–3.347, *p* = 0.03842) were found to potentially promote the onset of SCLC.

**Conclusion:**

We identified potential causal relationships between certain gut microbiota and SCLC, offering new insights into microbiome‐mediated mechanisms of SCLC pathogenesis, resistance, mutations, and more.

## Graphical abstract

Mendelian randomization to investigate the causal impact of the gut microbiota on small cell lung cancer (SCLC). It begins with the careful selection of genetic instruments linked to the microbiome, utilizes robust statistical methods to address confounding elements, and arrives at meaningful conclusions. The findings are particularly significant, revealing that specific genera and families within the gut microbiota may play a role in either increasing the risk or offering protection against SCLC. This research sheds light on the intricate relationship between the gut microbiome and lung cancer, opening new avenues for understanding and potentially intervening in SCLC development.

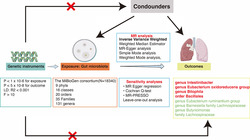

## 1 INTRODUCTION

Lung cancer, as a malignant disease, boasts alarmingly high mortality and morbidity rates worldwide.^1^ According to global cancer statistics, it is estimated that over 20 000 individuals are newly diagnosed with lung cancer annually.^2^, ^3^ Particularly noteworthy is the fact that approximately half of these new lung cancer cases occur in Asia.^4^ In China, lung cancer has been the leading cause of death among all malignant tumors since 2011.^5^ Small cell lung cancer (SCLC), one of the primary types of lung cancer, is a high‐grade neuroendocrine carcinoma predominantly found in smokers. Moreover, compared to other lung cancer types, SCLC has a notably poor prognosis.^6^ While smoking is the primary risk factor for SCLC, other risk factors such as exposure to asbestos, radiation, and environmental pollution should not be overlooked.^1^, ^7^, ^8^

The microbiome is increasingly recognized as a pivotal player in cancer onset, progression, and response to chemotherapy. In recent years, both preclinical and clinical studies have established a connection between the microbiome and lung cancer.^9^ The gut and lungs share a common embryonic origin,^10^ and there is physical interaction between them, as ingested microbes can enter the gastrointestinal and respiratory tracts and gastroesophageal contents can be aspirated into the lungs.^11^ Due to this extensive dialogue between the gut and lungs, research targeting the gut–lung axis (GLA) has become paramount in disease studies in recent years.^12^ However, compared to other diseases like Crohn's disease and colitis, our understanding of how the microbiome affects the lungs remains limited. A study investigating female non‐smoking lung cancer patients revealed correlations between the gut microbiome and TNM staging and primary tumor size.^13^ Specifically, a significant positive correlation was found between the relative abundance of *Faecalibacterium* and primary tumor size, while a significant negative correlation was observed with *Clostridium* and *Pseudomonas*. Another study suggested that lung cancer patients have lower concentrations of Firmicutes and Proteobacteria and relatively higher levels of *Pseudomonas* and *Clostridium* compared to healthy individuals.^14^ Given the vast individual variations in gut microbiota, it is unsurprising to observe inconsistencies in the same microbial communities across different lung cancer patients. It is crucial to note that these studies were observational, conducted on clinical samples. The observed changes might be attributed to the effects of bacterial metabolic by‐products or molecules on the immune system. For instance, in individuals with existing tumors, *Pseudomonas* and *Faecalibacterium* might suppress tumor proliferation by activating T ncells.^15^ However, whether these microbial communities exert similar anti‐tumor effects in tumor‐free individuals remains uncertain. Moreover, in observational studies, the association between gut microbiota and SCLC can easily be confounded by factors like age, environment, dietary patterns, and lifestyle, which are challenging to control effectively. These conditions limit the causal inference between the gut microbiome and SCLC.

In this study, we employ Mendelian randomization (MR) as an innovative methodology to delve into the potential causal linkage between the gut microbiome and SCLC.^16^ MR capitalizes on genetic variations to formulate instrumental variables (IVs) for exposures, thereby seeking to elucidate the causal nexus between these exposures and disease manifestations.^17^ Since genetic variances are inherently determined, their associations with outcomes are insulated from prevalent confounders, rendering MR particularly suited for discerning authentic causal connections. The MR approach has been prolifically harnessed to investigate the causal interplay between the gut microbiome and a spectrum of ailments, encompassing gynecological maladies,^18^ autoimmune disorders,^16^, ^19^ and metabolic syndromes.^20^ Drawing upon the genome‐wide association study (GWAS) summary data from the MiBioGen and FinnGen consortia, we embarked on a unidirectional MR evaluation to gauge the causal interrelation between the gut microbiome and SCLC.

## 2 MATERIALS AND METHODS

### 2.1 Data sources

Genetic variation data for the gut microbiome were obtained from the most extensive gut microbiome composition genome‐wide meta‐analysis published by the MiBioGen consortium.^21^ This study encompasses 16SrRNA gene sequencing spectra and genotyping data from 18 340 individuals across 24 countries, including the United States, the United Kingdom, Finland, Sweden, Denmark, and the Netherlands. The summarized data from this research includes bacteria from nine phyla, 16 classes, 20 orders, 35 families, and 131 genera. Statistical data for SCLC were sourced from the FinnGen consortium.^22^ This GWAS comprises 218 792 European adult participants, with 179 cases and 218 613 controls.

### 2.2 Selection of IVs

**FIGURE 1:**
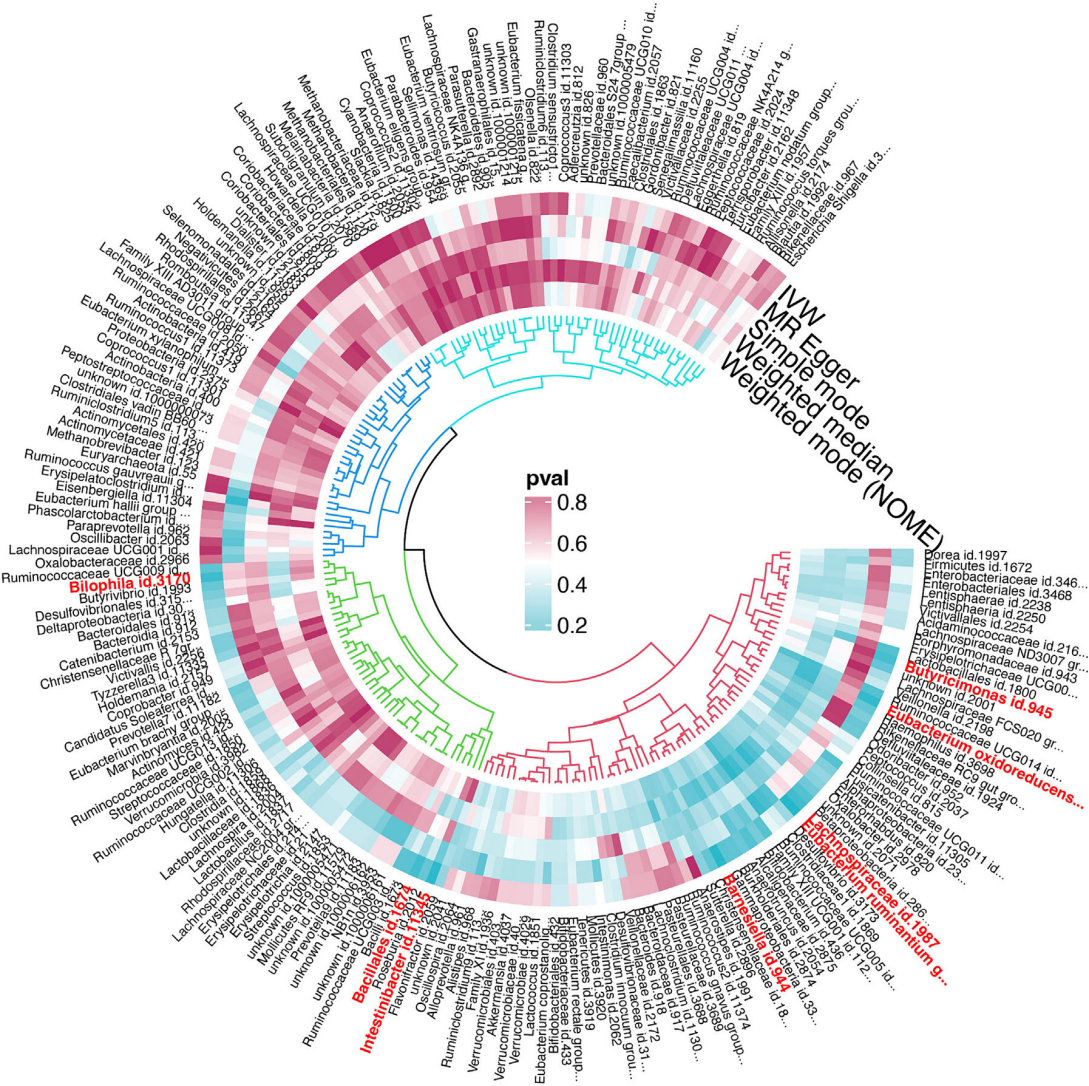
All results of Mendelian randomization (MR) analysis and sensitivity analysis between gut microbiota and small cell lung cancer (SCLC).

With the gut microbiome as the exposure and SCLC as the outcome, the IVs, that is, single nucleotide polymorphisms (SNPs), must satisfy three critical assumptions in Figure 1, ^23^:

1. Relevance assumption: The IV is strongly correlated with the exposure.
2. Exclusion restriction assumption: The IV is not related to the outcome.
3. Independence assumption: The IV is not associated with confounders that could lead to the outcome.

Consistent with many contemporary MR studies,^24^ we employed a genome‐wide significance threshold of *p* < 5 × 10^−8^ for SNP selection. Due to the scarcity of SNPs reaching this stringent threshold, a more lenient threshold of *p* < 5 × 10^−6^ was adopted for potential IVs for each exposure of interest. To ensure IV independence, we instituted linkage disequilibrium clumping using a clumping window of 10 MB and an *R* ^2^ threshold of <0.001, referencing European ancestry data from the 1000 Genomes Project. To mitigate bias from potentially weak instruments, we computed *F*‐statistics for each SNP, retaining only those deemed as strong IVs (*F*‐statistics > 10). Additionally, ambiguous and palindromic SNPs, which could not be rectified during harmonization, were systematically excluded.

### 2.3 Statistical analysis

We employed the inverse variance weighted (IVW) method as the primary analysis for MR. Additionally, four other methods, including the weighted median estimator (WME), MR‐Egger analysis, simple mode analysis, and weighted mode analysis, were applied as supplementary references. The IVW method provides consistent estimates only when all SNPs serve as valid IVs, without considering the presence of an intercept term, implying no pleiotropy for all IVs.^25^, ^26^ The WME is based on the assumption that over half of the SNPs are valid IVs.^27^ In contrast, the MR‐Egger method assumes all SNPs are invalid IVs, defaulting to the presence of an intercept term.^28^ When IVW results are significant, further sensitivity analyses are incorporated. For exposures with only one IV, the Wald ratio method is employed to estimate the effect of exposure on the outcome.

Sensitivity analyses encompassed heterogeneity and pleiotropy tests. The MR‐Egger intercept test detects horizontal pleiotropy, where its intercept represents potential pleiotropy, and its *p*‐value validates the significance of this pleiotropy.^29^ Cochran's Q test examines the heterogeneity of IVs, with a *p*‐value greater than 0.05 indicating no heterogeneity.^30^ Moreover, we employed the “leave‐one‐out analysis” to determine if a significant association is driven by a single SNP, identifying potential outlier SNPs. Subsequent to this, SNPs exhibiting pleiotropy or heterogeneity were identified and removed based on the leave‐one‐out results.

Statistical *F*‐statistics were computed using the formula $F=\frac{R^{2}\;x\;\left(n-k-1\right)}{\left(1-R^{2}\right)\;x\;k}$,^31^ where *n* represents the sample size, *k* represents the number of IVs, and *R* ^*2*^ represents the proportion of variance in the exposure explained by genetic variation. When evaluating a single IV, k = 1. An *F*‐statistic > 10 for a single SNP indicates no significant weak instrument bias; otherwise, that IV should be excluded.

After excluding the aforementioned non‐compliant IVs, we repeated the MR analysis to obtain the final MR estimates. In the absence of heterogeneity and pleiotropy, IVW serves as a more reliable fitting model.^28^ For binary outcomes, effect estimates are presented as odds ratios (OR) with 95% confidence intervals.

MR analyses were conducted in the R computational environment (version 4.2.3) using the TwoSampleMR package (version 0.5.6). A *p*‐value < 0.05 was considered statistically significant evidence of a causal effect.

## 3 RESULTS

### 3.1 IV selection and preliminary MR analysis

Based on our IV selection criteria, we identified 2426 SNPs as instruments for examining 211 distinct gut microbiota taxa. Each selected SNP demonstrated an *F*‐statistic greater than 10, indicating a robust association with the microbiota taxa and minimizing the concern of weak instrument bias in our analysis. The relationships between these 211 bacterial taxa and SCLC, facilitated by the chosen SNPs, are visually represented in Figure 1. For an in‐depth review of the SNPs utilized as IVs, including their association strengths and other relevant statistics, please refer to Table S1. Moreover, the comprehensive results of the MR analysis, detailing the influence of each microbiota taxon on SCLE risk, are systematically compiled in Table S2.

### 3.2 Detailed MR analysis

**FIGURE 2:**
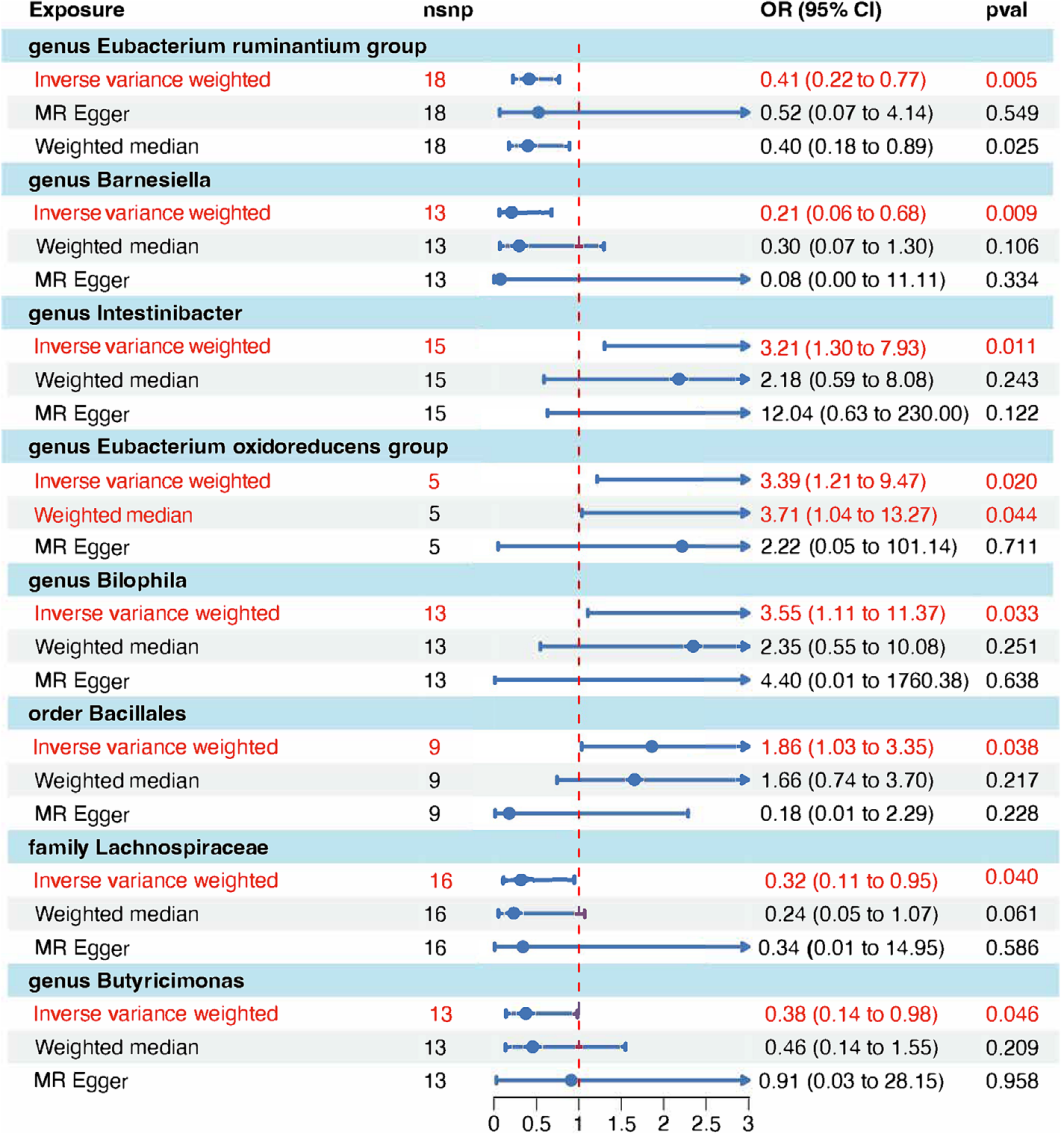
Forest plot of causal relationships estimated for gut microbiome and small cell lung cancer (SCLC) using the inverse variance weighted method.

The results of our MR analysis, depicted in Figure 2, demonstrate that the IVW method identified significant associations between five bacterial taxa and SCLC. To ensure the robustness of these findings, we further employed the WME and MR‐Egger methods. While significant associations were observed with the IVW method, the analyses conducted using the WME and MR‐Egger methods did not yield statistically significant results for the taxa under investigation. This variation in outcomes across different MR methods might indicate underlying heterogeneity or pleiotropy within the IVs selected for our study. The divergent results from these methods illuminate the intricate role gut microbiota may play in influencing SCLC, emphasizing the necessity of employing a multifaceted MR approach to corroborate causal relationships. Specifically, the genus *Eubacterium ruminantium* group (OR = 0.413, 95% CI: 0.223–0.767, *p* = 0.00513), genus *Barnesiella* (OR = 0.208, 95% CI: 0.0640–0.678, *p* = 0.00919), family Lachnospiraceae (OR = 0.319, 95% CI: 0.107–0.948, *p* = 0.03979), and genus *Butyricimonas* (OR = 0.376, 95% CI: 0.144–0.984, *p* = 0.04634) were found to have a protective effect against SCLC.

Conversely, the results for the genus *Intestinibacter* (OR = 3.214, 95% CI: 1.303–7.926, *p* = 0.01125), genus *Eubacterium oxidoreducens* group (OR = 3.391, 95% CI: 1.215–9.467,*p* = 0.01973), genus *Bilophila* (OR = 3.547, 95% CI: 1.106–11.371, *p* = 0.03315), and order Bacillales (OR = 1.860, 95% CI: 1.034–3.347, *p* = 0.03842) indicated the opposite. These taxa might serve as potential inducers or risk factors for the development of SCLC.

### 3.3 Sensitivity analysis

The results of the sensitivity analysis are presented in Table 1. Cochran's Q test revealed that the test values for six bacterial genera were all greater than 0.05, indicating that there is no heterogeneity among the IVs for these taxa. Furthermore, the differences between the intercepts from the MR‐Egger method and zero were not significant, confirming the absence of horizontal pleiotropy in the results. Scatter plots, as depicted in Figure S1, highlight the effect trends across different MR methods without any significant outliers. Concurrently, the leave‐one‐out analysis, presented in Figure S2, underscores the data's robustness, indicating that no individual SNP had a disproportionate impact on the overall findings. This further solidifies the reliability of our MR results.

**TABLE 1:** Mendelian randomization sensitivity analysis of the influence of gut microbiota on small cell lung cancer risk.

No.	Bacterial taxa	MR‐Egger		IVW		Horizontal pleiotropy	
		*Q*	*p‐value*	*Q*	*p‐value*	*Egger_intercept*	*p‐value*
1	Family Lachnospiraceae	3.07	0.878	6.50	0.592	−0.004	0.971
2	Genus *Barnesiella*	15.3	0.170	15.5	0.215	0.077	0.694
3	Genus *Bilophila*	15.2	0.175	15.2	0.232	−0.016	0.944
4	Genus *Butyricimonas*	4.54	0.952	4.81	0.964	−0.079	0.61
5	Genus *Eubacterium oxidoreducens* group	0.608	0.895	0.659	0.956	0.047	0.835
6	Genus *Eubacterium ruminantium* group	9.91	0.871	9.97	0.905	−0.024	0.815
7	Genus *Intestinibacter*	14.0	0.373	14.9	0.393	−0.111	0.373
8	Order Bacillales	3.07	0.878	6.50	0.592	0.361	0.107

## 4 DISCUSSION

In this study, we employed MR to analyze 211 prevalent microbial taxa in the gut. Our findings suggest that four bacterial taxa, namely, the genus *E. ruminantium* group, genus *Barnesiella*, family Lachnospiraceae, and genus *Butyricimonas*, may have protective effects against SCLC. Conversely, the genera *Intestinibacter*, *E. oxidoreducens* group, *Bilophila*, and the order Bacillales might contribute to the onset of SCLC.

Previous studies have suggested that the gut microbiota of lung cancer patients differs significantly from that of healthy individuals.^32^, ^33^, ^34^, ^35^, ^36^ Moreover, the use of antibiotics or probiotics as adjunctive treatments for lung cancer, especially in conjunction with immunotherapy or mutation prevention, underscores the close association between alterations in the gut microbiome and lung cancer. However, due to the inherent limitations of observational studies and animal experiments, these studies are insufficient to determine the specific roles of different gut microbial taxa in lung cancer. Notably, current research is predominantly focused on non‐small cell lung cancer (NSCLC), with scant studies on SCLC, which also has a high incidence rate. We conducted a comprehensive systematic analysis of 211 taxa of the gut microbiome, identifying specific bacteria that may have beneficial or detrimental effects on SCLC patients. This contributes to a deeper understanding of the pathogenesis of SCLC and offers potential avenues for novel clinical treatment strategies.^37^ In a previous study, it was also observed^38^ that patients with SCLC exhibited a significant decrease in the abundance of the family Lachnospiraceae in their gut microbiota composition. Our research corroborates this finding, suggesting that family Lachnospiraceae might be one of the protective factors against SCLC. Earlier studies have also indicated that the Spirochaetaceae family can protect the host from cancer by producing butyrate.^39^ This could potentially explain the protective mechanism of family Lachnospiraceae against SCLC. Moreover, for the first time, we identified that the genus *Butyricimonas* might act as a protective factor for SCLC. Given that genus *Butyricimonas* is also a butyrate‐producing bacterium, we speculate that it might share a similar mechanism with Spirochaetaceae, exerting antitumor effects through the anti‐inflammatory properties of butyrate.^40^ However, this hypothesis warrants further investigation for validation.

On the other hand, we found that the genus *Intestinibacter*, genus *Bilophila*, and order Bacillales might promote the onset of SCLC. The genus Intestinibacter has previously been associated with NSCLC.^41^, ^42^ Its abundance is notably increased in the gut microbiota of NSCLC patients. Our study further suggests a causal link between this bacterial group and the occurrence of SCLC. We hypothesize that this might be achieved by recruiting myeloid‐derived suppressor cells, tumor‐associated macrophages, and regulatory T cells to inhibit the anti‐tumor immune response, leading to the development of SCLC.^43^ As for the genus *Bilophila*, there are no direct reports linking it to tumor development. However, in a study on PD‐1 treatment, it was observed that its abundance significantly increased in non‐responders, indicating that it might not be beneficial in lung cancer patients. We speculate that since the genus *Bilophila* is a hydrogen sulfide‐producing bacterium and hydrogen sulfide is a genotoxic compound,^44^ it has been proven to damage DNA, leading to genomic or chromosomal instability. This might be one of the reasons it promotes the onset of SCLC or tumor mutations. A pilot study suggested that the abundance of order Bacillales increases in lung cancer patients, a finding that our research also confirms.^45^ We hypothesize that the increased number of order Bacillales might allow endotoxins to continuously enter the circulation through a compromised gut barrier, triggering a systemic inflammatory response, which in turn promotes tumor development.^46^ Targeting these harmful bacterial groups therapeutically might help reduce the incidence of SCLC or enhance the therapeutic effects against SCLC.

Our research has identified some beneficial and harmful bacterial groups involved in the pathogenesis of SCLC. However, this evaluation is based on causality, and further research is needed in the future to clarify its mechanism. It is also worth noting that our study was conducted at the genus level of the entire microbial community. Different species of bacteria within the same genus may have different pathological or physiological effects, and we need to be aware of the heterogeneity that exists within them. As we found in our study, the genus *E. ruminantium* group plays a protective role against SCLC, while the genus *E. oxidoreducens* group from the same genus might promote the onset of SCLC. Both of these bacterial groups are Firmicutes, which have been extensively studied and shown to undergo significant changes in lung cancer patients,^47^, ^48^, ^49^ but their mechanisms are still not fully understood. On one hand, an increase in Firmicutes has been linked to increased gut permeability and chronic inflammation.^50^, ^51^ The expression of inflammatory factors such as interleukin (IL)‐6 and IL‐1b increases, which may promote the onset of SCLC through pro‐inflammatory effects. On the other hand, the abundance of *Eubacterium* in the gut is related to the level of short‐chain fatty acids (SCFAs), and high levels of SCFAs have beneficial effects on tumors under clinical conditions.^47^ Therefore, it is not surprising that different species of bacteria from the same genus have different effects in SCLC.

In summary, the complex interactions among gut microbiota might explain the discrepancies between genetic prediction results and clinical observations. We speculate that this is largely due to the involvement of gut microbes in inflammation and immune regulation, thereby participating in the entire pathophysiological process of SCLC. However, further prospective randomized controlled trials are needed to validate our conclusions. Specifically, future research should explore the potential of gut probiotic interventions or antibiotic treatments for SCLC, especially when used in combination with immune checkpoint inhibitors. There are certain limitations in our study. Firstly, although our research analyzed common gut microbial communities, the gut microbiota is vast and highly heterogeneous. Moreover, our study only analyzed populations from Europe, which means that caution is needed when extrapolating our findings to individuals of other ethnicities.

## 5 CONCLUSION

In summary, our research findings support the notion that the bacterial taxa genus *E. ruminantium* group, genus *Barnesiella*, family Lachnospiraceae, and genus *Butyricimonas* may have protective effects against SCLC. On the other hand, the bacterial taxa genus *Intestinibacter*, genus *E. oxidoreducens* group, genus *Bilophila*, and order Bacillales might promote the development of SCLC. It is particularly noteworthy that the genus *Eubacterium* might have dual effects on human health, with specific species and strains potentially having different impacts. Therefore, further research is needed to elucidate the potential mechanisms of these bacterial taxa in the context of SCLC.

## AUTHOR CONTRIBUTIONS

Wenjing Yang formulated the research questions and design, conducted the analysis, and drafted the manuscript for submission; Yan Chen and Wangshu Li contributed to drafting the submitted manuscript; Xinxia Fan assisted in data analysis and performed statistical analysis. All authors have read and agreed to the published version of the manuscript.

## CONFLICT OF INTEREST STATEMENT

The authors declare no potential conflict of interest.

## ETHICS STATEMENT

The study leverages GWAS data that are openly accessible and have been de‐identified. These data have previously received approval from the Institutional Review Board (IRB), obviating the need for further ethical clearance.

## Supporting information


**Figure S1.** Scatter plots of the causal effects of gut microbiota on the risk of SCLC. *family Lachnospiraceae; (B)genus Barnesiella; (C)genus Butyricimonas; (D)order Bacillales; (E)genus Intestinibacter; (F)genus Bilophila; (G)genus Eubacterium oxidoreducens group; (H)genus Eubacterium ruminantium group.*

**Figure S2.** Leave‐one‐out sensitivity analyses of the causal effects of gut microbiota on the risk of SCLC. family Lachnospiraceae; (B)genus Barnesiella; (C)genus Butyricimonas; (D)order Bacillales; (E)genus Intestinibacter; (F)genus Bilophila; (G)genus *Eubacterium oxidoreducens* group; (H)genus *Eubacterium ruminantium* group.

**Data S1.** Supplementary Material.

**Table S1.** Exposure:GWAS summary data of bacterial taxa from the MiBioGen consortium at P‐value<5.0 × 10–6.

**Table S2.** The estimates of two‐sample MR of gut microbiome and SCLC.

## Data Availability

The data that support the findings of this study are available in GWAS at https://www.ncbi.nlm.nih.gov/gap. These data were derived from the following resources available in the public domain: https://www.ebi.ac.uk/gwas/.
