# Supplementary figures and images for: Causal influence of gut microbiota on small cell lung cancer: a Mendelian randomization study
Source: Clin Respir J. 2024 Apr 29;18(5):e13764. doi: 10.1111/crj.13764 (PMC11058399; doi:10.1111/crj.13764)

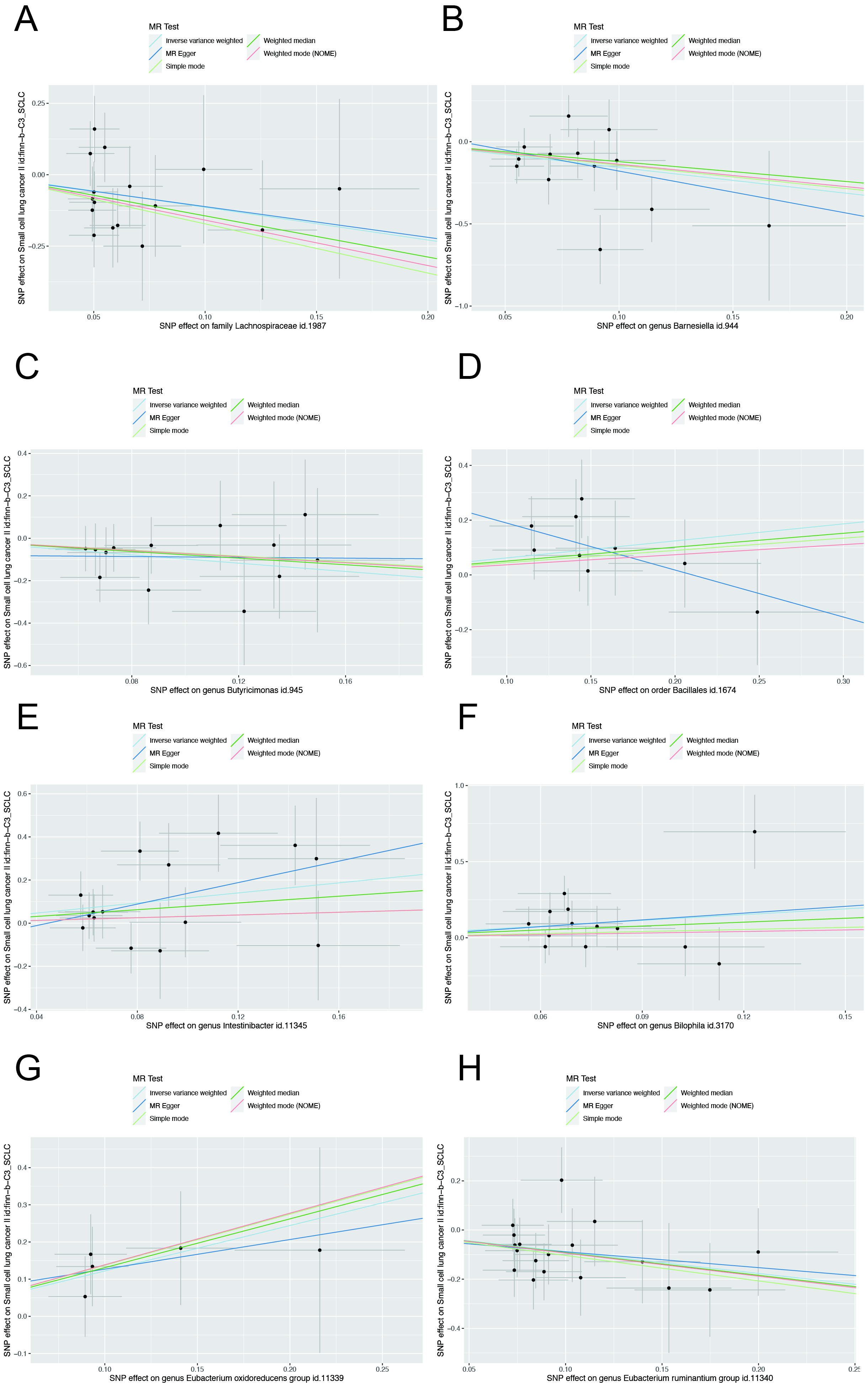

Supplement: Supplementary file 1 — Figure S1. Scatter plots of the causal effects of gut microbiota on the risk of SCLC. family Lachnospiraceae; (B)genus Barnesiella; (C)genus Butyricimonas; (D)order Bacillales; (E)genus Intestinibacter; (F)genus Bilophila; (G)genus Eubacterium oxidoreducens group; (H)genus Eubacterium ruminantium group. [file CRJ-18-e13764-s002.tif]

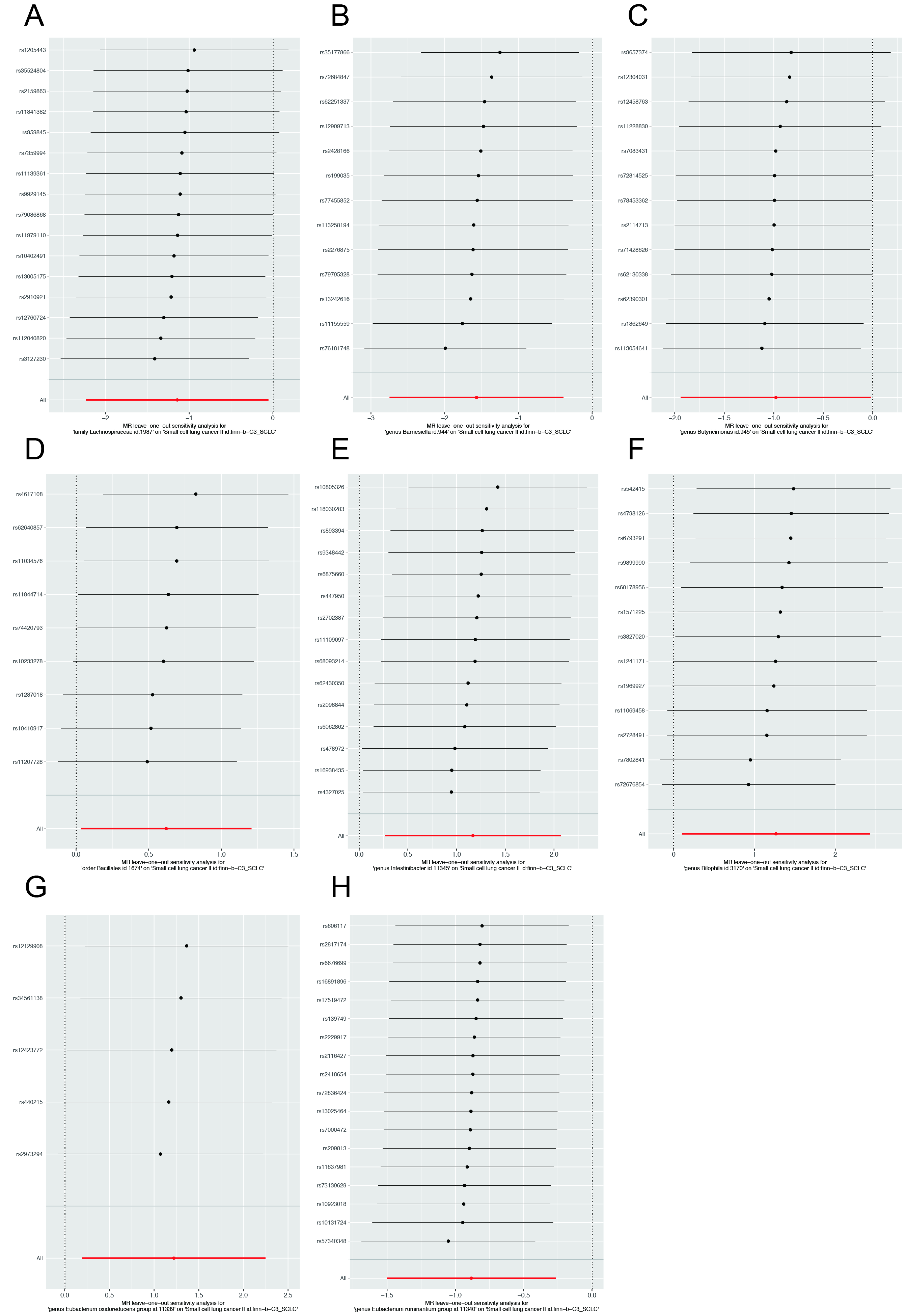

Supplement: Supplementary file 2 — Figure S2. Leave‐one‐out sensitivity analyses of the causal effects of gut microbiota on the risk of SCLC. family Lachnospiraceae; (B)genus Barnesiella; (C)genus Butyricimonas; (D)order Bacillales; (E)genus Intestinibacter; (F)genus Bilophila; (G)genus Eubacterium oxidoreducens group; (H)genus Eubacterium ruminantium group. [file CRJ-18-e13764-s004.tif]
