# Supplementary material for: Causal influence of gut microbiota on small cell lung cancer: a Mendelian randomization study
Source: Clin Respir J. 2024 Apr 29;18(5):e13764. doi: 10.1111/crj.13764 (PMC11058399; doi:10.1111/crj.13764)
Supplement: Supplementary file 3 — Data S1. Supplementary Material. [file CRJ-18-e13764-s005.docx]

Supplementary Figures

**Supplementary Figure S1** Scatter plots of the causal effects of gut microbiota on the risk of SCLC.

1. *family Lachnospiraceae ; (B)genus Barnesiella; (C)genus Butyricimonas; (D)order Bacillales; (E)genus Intestinibacter; (F**)genus Bilophila; (G**)genus Eubacterium oxidoreducens group; (H**)genus Eubacterium ruminantium group.*


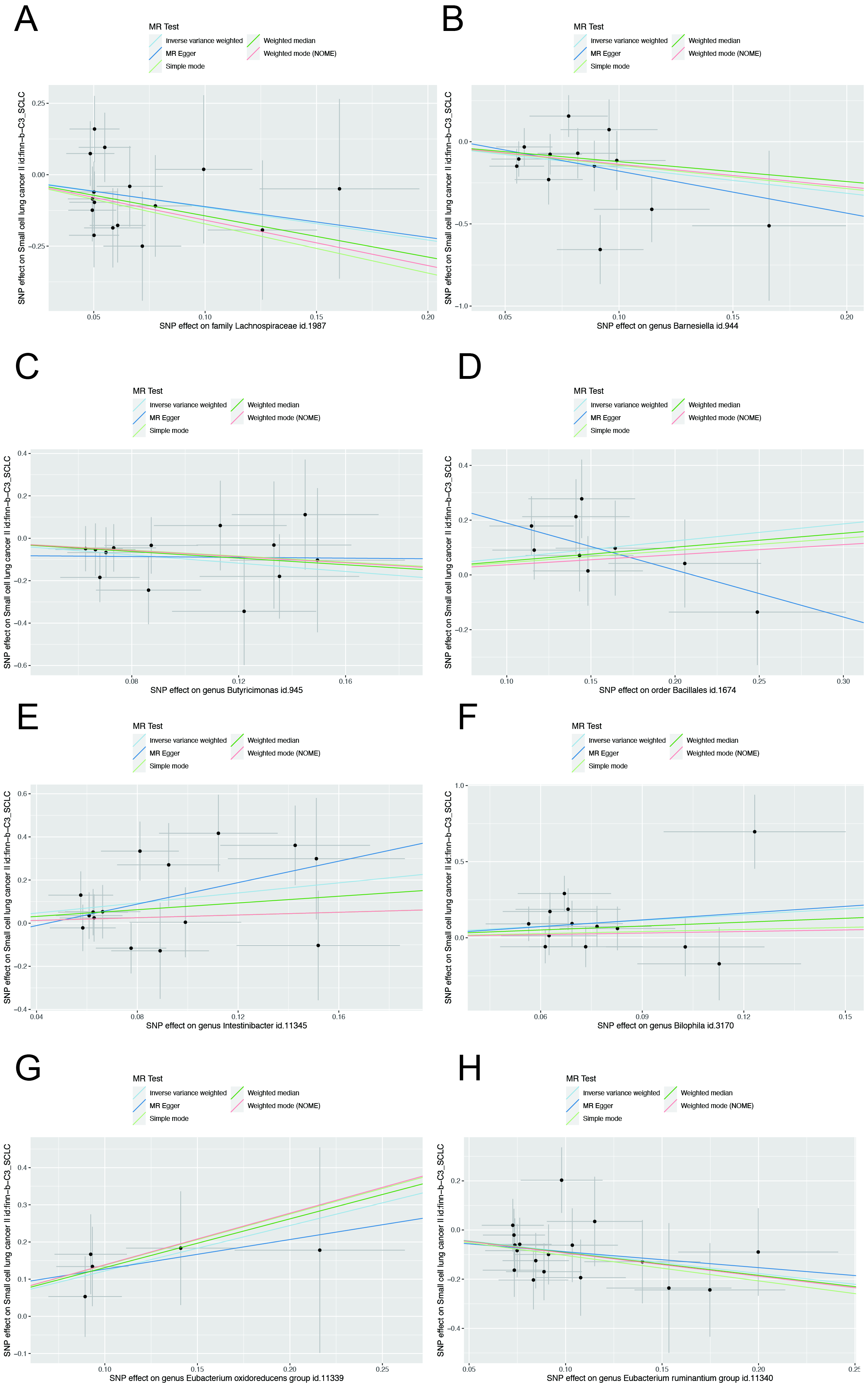


**Supplementary Figure S2** Leave-one-out sensitivity analyses of the causal effects of gut microbiota on the risk of SCLC.

1. *family Lachnospiraceae ; (B)genus Barnesiella; (C)genus Butyricimonas; (D)order Bacillales; (E)genus Intestinibacter; (F)genus Bilophila; (G)genus Eubacterium oxidoreducens group; (H)genus Eubacterium ruminantium group.*

*
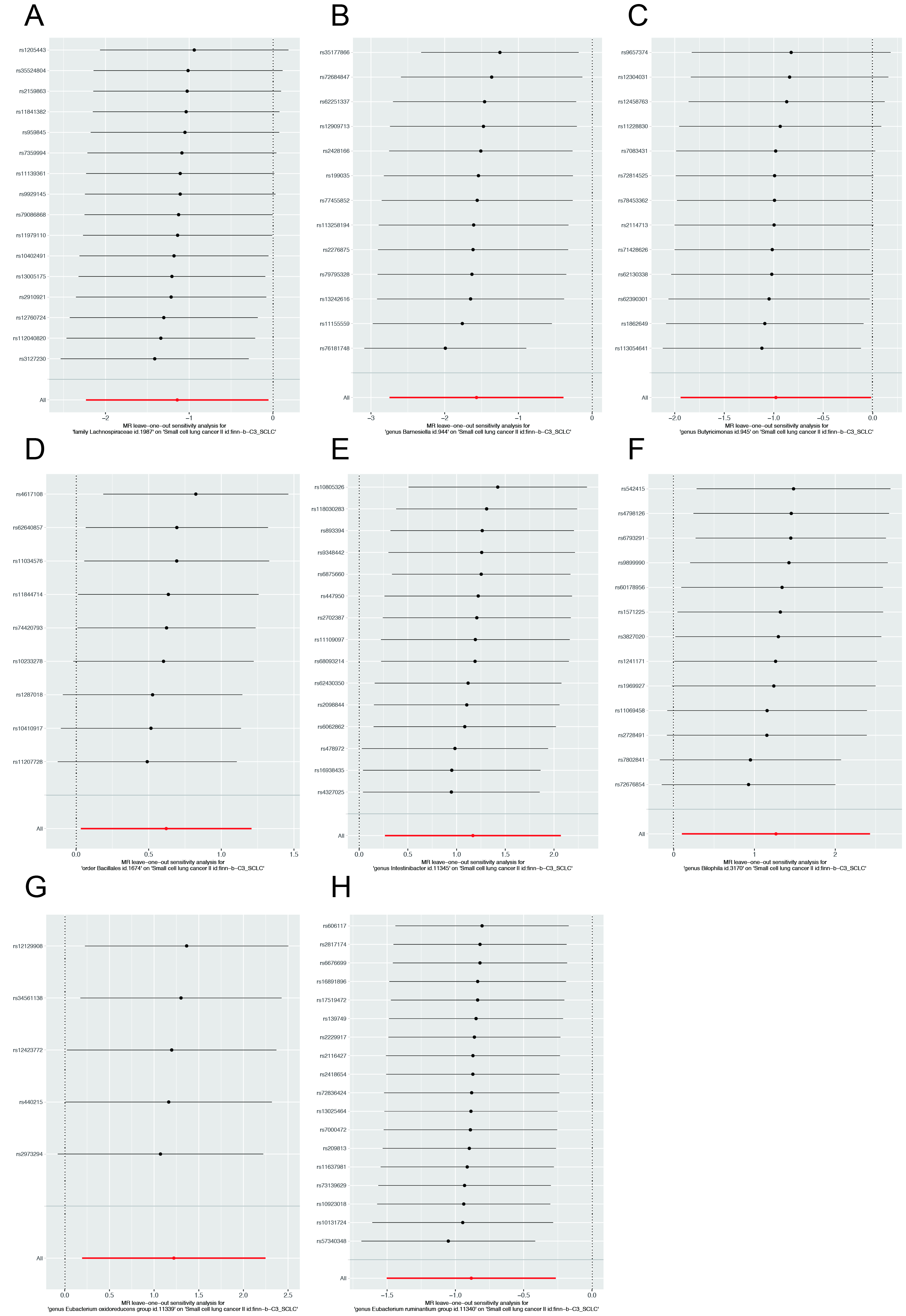
*
